# Monitoring of kinetics and exhaustion markers of circulating CAR-T cells as early predictive factors in patients with B-cell malignancies

**DOI:** 10.3389/fimmu.2023.1152498

**Published:** 2023-04-14

**Authors:** Clara Beatriz García-Calderón, Belén Sierro-Martínez, Estefanía García-Guerrero, Luzalba Sanoja-Flores, Raquel Muñoz-García, Victoria Ruiz-Maldonado, María Reyes Jimenez-Leon, Javier Delgado-Serrano, Águeda Molinos-Quintana, Beatriz Guijarro-Albaladejo, Inmaculada Carrasco-Brocal, José-Manuel Lucena, José-Raúl García-Lozano, Cristina Blázquez-Goñi, Juan Luis Reguera-Ortega, María-Francisca González-Escribano, Marta Reinoso-Segura, Javier Briones, José Antonio Pérez-Simón, Teresa Caballero-Velázquez

**Affiliations:** ^1^ Servicio de Hematología, Hospital Universitario Virgen del Rocío, Instituto de Biomedicina de Sevilla, (IBIS/CSIC), Universidad de Sevilla, Sevilla, Spain; ^2^ Servicio de Inmunología, Instituto de Biomedicina de Sevilla (IBiS), Hospital Universitario Virgen del Rocío, Centro Superior de Investigaciones Científicas (CSIC), Universidad de Sevilla, Sevilla, Spain; ^3^ Hematology Service, Hospital de la Santa Creu i Sant Pau, Barcelona, Spain

**Keywords:** CAR-T, flow cytometry, dPCR (digital PCR), monitoring, biomarkers, B-ALL, Lymphoma

## Abstract

**Purpose:**

CAR-T cell therapy has proven to be a disruptive treatment in the hematology field, however, less than 50% of patients maintain long-term response and early predictors of outcome are still inconsistently defined. Here, we aimed to optimize the detection of CD19 CAR-T cells in blood and to identify phenotypic features as early biomarkers associated with toxicity and outcomes.

**Experimental design:**

In this study, monitoring by flow cytometry and digital PCR (dPCR), and immunophenotypic characterization of circulating CAR-T cells from 48 patients treated with Tisa-cel or Axi-cel was performed.

**Results:**

Validation of the flow cytometry reagent for the detection of CAR-T cells in blood revealed CD19 protein conjugated with streptavidin as the optimal detection method. Kinetics of CAR-T cell expansion in blood confirmed median day of peak expansion at seven days post-infusion by both flow cytometry and digital PCR. Circulating CAR-T cells showed an activated, proliferative, and exhausted phenotype at the time of peak expansion. Patients with increased expansion showed more severe CRS and ICANs. Immunophenotypic characterization of CAR-T cells at the peak expansion identified the increased expression of co-inhibitory molecules PD1 and LAG3 and reduced levels of the cytotoxicity marker CD107a as predictors of a better long-term disease control.

**Conclusions:**

These data show the importance of CAR-T cells in vivo monitoring and identify the expression of PD1LAG3 and CD107a as early biomarkers of long-term disease control after CAR-T cell therapy.

## 1 Introduction

Adoptive immunotherapy using engineered chimeric antigen receptor (CAR)-T cells has stood out as a disruptive treatment in relapsed/refractory B cell leukemia and lymphoma. Despite the outstanding results of CAR-T therapy in patients with B-cell malignancies, less than 50% of patients experience long-term disease response (1). Therefore, intensive efforts are being made to improve the outcomes of these patients and to identify biomarkers capable to early predict patient outcomes (2).

Among other factors, the efficacy of the treatment has been related to specific features of the CAR-T cell product as well as CAR-T expansion and persistence after infusion. Accordingly, some trials have shown a correlation between *in vivo* expansion and persistence of CAR-T cells in peripheral blood of pediatric B-ALL patients with response, event free-survival and the incidence of cytokine release syndrome (CRS) or immune effector cell-associated neurotoxicity syndrome (ICANS) (3–7). However, some other trials have shown inconsistent or weak correlation between CAR-T expansion and clinical outcomes (8–10). Therefore, this topic is still an area of debate and further studies are required to gain more insight into the role of CAR-T monitoring as a predictive biomarker. Furthermore, the analysis of leukapheresis products has shown that an elevated frequency of CD27+CD45RO-CD8+ T cells and a higher CD4+ to CD8+ T cells ratio correlated with a higher complete response (CR) (6, 11). In addition, RNA-seq analyses of CD19 CAR-T cell products has revealed differences in gene expression in T cell differentiation and metabolism from responder and non-responder patients (6, 12). Increased expression of the co-inhibitory receptors PD1+, TIM3+ and LAG3+ in the leukapheresis and the infusion product has also been associated with treatment failure in several studies (6, 12, 13).

On the other side, scanty information is available regarding the functional and/or immunophenotypic characteristics of the circulating CAR-T cells after infusion. Recently, Good, et al., have discovered a correlation between increased levels of CAR-Treg population at day 7 post-infusion and progression at 6 months in lymphoma patients treated with Axicabtagene ciloleucel. Besides, this population has also been associated with reduced neurotoxicity (8). Nevertheless, the information according to immunophenotypic characteristics of CAR-T cells post-infusion is limited and whether these features might have any predictive value in the long term has not been clearly elucidated.

The persistence and monitoring of circulating CAR-T cells in peripheral blood of patients can be followed by molecular techniques such as quantitative or digital polymerase chain reaction (PCR), being the digital PCR more sensitive and precise to detect CAR-T cells (14–16), and/or by flow cytometry. The identification and characterization of CAR-T cells by flow cytometry can be very challenging since its detection may depend on the cell product. Several reagents are commercially available for the detection of CAR-T cells (11, 17). Also, indirect methods have also been tested for the detection of CAR-T cells such as Protein L that binds to kappa light chain of immunoglobulins (18). Additionally, some CAR constructs have incorporated detection markers to facilitate *in vivo* monitoring (2). Thus, validation of all these detection methods should be done for each CAR-T cell product prior *in vivo* monitoring.

In this study, we propose the validation of a sensitive and specific method for the identification of CD19 CAR-T cells by flow cytometry. Samples from forty-eight patients receiving commercial CD19 CAR-T cells for B-ALL or DLBCL treatment were analyzed by flow cytometry and by digital PCR up to 20 days post-infusion. Finally, correlation studies between the cell kinetics and immunophenotype characterization of circulating CAR-T cells post-infusion were performed in an attempt to search for early biomarkers associated with a better response, thus allowing to identify those groups of patients with worse prognosis at very early time points after infusion, when therapeutic maneuvers might have a higher probability of success before relapse does occur.

## 2 Materials and methods

### 2.1 *Ex vivo* PBMCs isolation and manufacturing of academic CD19 CAR-T cells

Peripheral blood mononuclear cells (PBMCs) were obtained from buffy coats of healthy donors kindly donated by the Regional Centre for Blood Transfusions at Virgen del Rocío University Hospital, Seville (Spain) after written informed consent. For academic CD19 CAR-T cell generation, CD4+ and CD8+ cells were isolated from human PBMCs, activated with anti-CD3/anti-CD28 (Gibco, Ref: 11131D) and transduced with a lentiviral vector containing CD19 CAR-T cell construct co-expressing EGFRt as a detection marker. Transduced cells were expanded with IL-2 (Miltenyi, Ref: 130-097-743) and irradiated feeder cells.

### 2.2 Patient samples

Samples were obtained from patients with relapsed/refractory acute lymphoblastic leukemia or lymphoma treated with CD19 CAR-T therapy (Tisagenlecleucel or Axicabtagene ciloleucel) at different time points: leukapheresis, days 5, 7, 11, 14 and 20 post CAR-T infusion.

### 2.3 Flow cytometry

Markers expression was evaluated by multicolor flow cytometry. For the validation of CAR-T detection of academic CD19 CAR-T, cells were thawed and washed with phosphate buffer saline (PBS) (Gibco, Ref: 18912-014). A quantity of 500,000 cells were used for each staining. To differentiate live/dead cells 7-AAD (BD Biosciences, Ref: 559925) was added to each sample. Samples were stained according to the antibodies and dyes shown in **Supplementary Table 1**.

For the monitoring of commercial CD19 CAR-T cells post-infusion, a quantity of 200,000 PBMCs was added for staining according to blood count. Samples were stained according to the antibodies and dyes shown in **Supplementary Table 1** and incubated 20 min at room temperature in the dark. Red blood cells were lysed using BD FACS Lysing solution (BD Biosciences, Ref: 349202) following mixing and 10 min incubation in the dark. Lysed cells were centrifuged at 2000 rpm 5 min and resuspended in FACs Flow solution (BD Biosciences, Ref: 12756528).

For the immunophenotypic characterization of the CAR-T, a bulk lysis of the sample was carried out according to the Euroflow protocol. Whole blood sample was lysed using ammonium chloride 1x (Cytognos, Ref: CYT-BCP-ALL-MRD-BL) and incubated in rotation for 15 min. Then, samples were washed twice with washing solution (PBS+0.2%BSA+ 1mM EDTA), centrifuged 10 min at 2000 rpm and resuspended in 100 uL FACs Flow solution (BD Biosciences, Ref: 12756528). Finally, cells were stained according to the antibodies and dyes shown in **Supplementary Table 1** and incubated 20 min at room temperature in the dark.

### 2.4 Digital PCR

To isolate peripheral blood mononuclear cells (PBMCs), Ficoll gradient with PD Vacutainer ^®^ CPT™ (Becton Dickinson, NY, USA) was used. To extract genomic DNA from PBMCs, the QIAmp DNA Mini Kit (Qiagen, Hilden, Germany) was used according to manufacturer’s recommendations. The purity and concentration of the DNA samples were measured using a Q3000 UV Spectrophotometer (Quawell, San Jose, CA, USA). Only samples with concentrations ≥20 ng/µl and an A260/280 absorbance ratio >1.8 were included in the study.

For Digital PCR (dPCR) reactions, the QuantStudio™ Absolute Q™ Digital PCR System (Applied Biosystems, CA, USA) was employed. To quantify CAR-T, primers and TaqMan probes designed to amplify PCR fragments in the FMC63 region of the CAR-T construct were used. The primers used were 5’-TGA AAC TGC AGG AGT CAG GA-3’ (forward) and 5’-CTG AGA CAG TGC ATG TGA CG-3’ (reverse), and the FAM TaqMan MGB probe was 5’-CTG GCC TGG TGG CGC CCT CA-3’. TaqMan^®^ Copy Number Reference Assay RNase P labelled with VIC (Applied Biosystems) was used as gen reference (REF). Each sample was simultaneously amplified to CAR-T and REF sequences using differently labelled probes. A 10 μL final volume mix reaction consisted of 80 ng of DNA, 2 μL of Absolute Q™ DNA dPCR Mix (5X), 250 nM of FMC63-probe, 900 nM of each FMC63-primer, and 0.5 μL primer-probe set for RNase P (20X). Cycle conditions were 96°C for 10 min, followed by 40 cycles at 96°C for 5 sec and 60°C for 15 sec.

### 2.5 Data analysis

No normalization was implemented in the flow cytometry data analysis. All raw data was analyzed with either FlowJo™ v10.7 Software (BD Life Sciences) or Infinicyt 2.0 (Cytognos) software. Statistical analyses were performed with GraphPad Prism v9 and SPSS 26. We applied unpaired two-sided Mann–Whitney U-tests to assess statistical significance between two groups of unpaired samples. We applied paired two-sided Wilcoxcon-matched-paired test to asses statistical significance between two groups of paired samples. AUC was calculated on days 5, 7, 11, 14 and 20 and peak CAR-T expansion was calculated only when data was obtained from at least three timepoints. For dPCR data analysis, “QuantStudio Absolute Q Digital PCR Software 6” (Applied Biosystems) with automatic Poisson correction was used. Normalization across samples was implemented by assuming there were two copies of the reference RNase P gen per cell. Results were expressed in CAR-T copies per cell. OS and EFS were estimated with the Kaplan-Meier method using survival in GraphPad Prism v9. EFS was computed from the date of infusion to the date of event, with both progression and death scored as event. To assess correlation, we calculated Spearman’s rank correlation coefficient.

## 3 Results

### 3.1 Validation of flow cytometric CD19 CAR staining with different reagents

**Figure 1:**
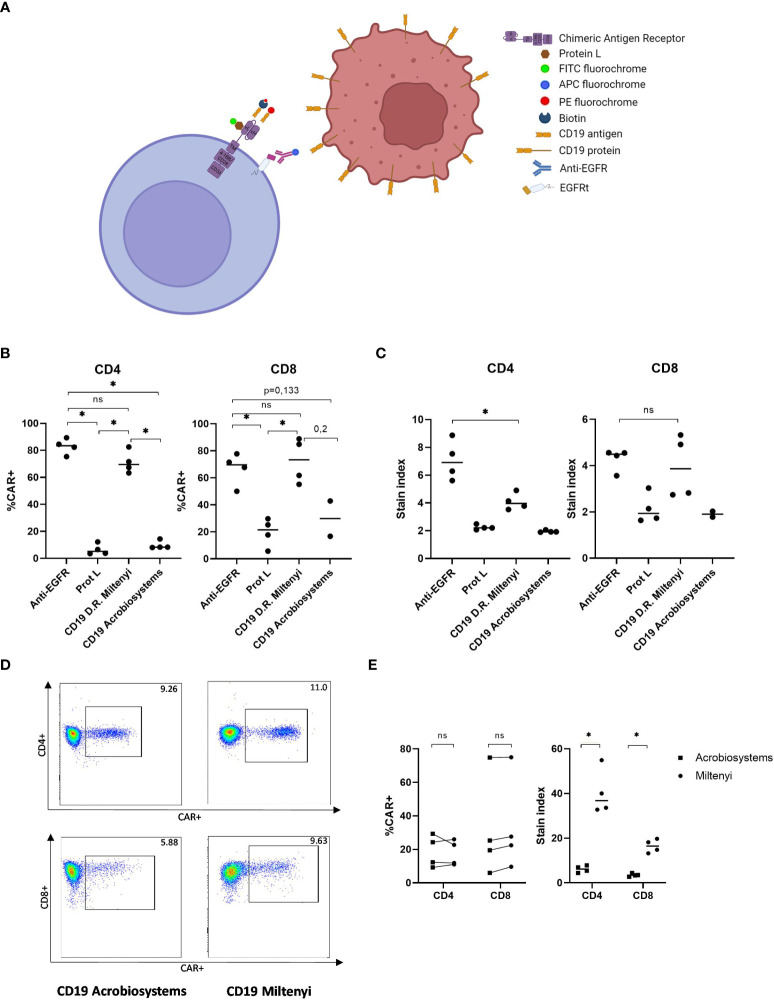
Validation of the detection reagent for academic and commercial CD19 CAR-T cells by flow cytometry. **(A)** Illustration of the different detection methods available for the identification of CAR-T cells by flow cytometry. **(B)** Percentage of detection of academic CD19 CAR-T cells in CD4 and CD8 compartments with different reagents. **(C)** Stain index of detection of academic CD19 CAR-T cells in CD4 and CD8 compartments with different reagents. **(D)** Example of flow cytometry plot of the identification of commercial CD19 CAR-T cells with two recombinant CD19 proteins. **(E)** Percentage *(left) and* stain index *(right)* of the detection of commercial CD19 CAR-T cells with two different reagents. Depicted are median and individual values of four independent experiments. P-values between the indicated groups were calculated using unpaired Mann-Whitney U-t tests. ns: non-significant, *p<0.05.

For the validation of CAR T- cell monitoring, distinct CAR T- cell detection methods using different reagents were performed. The binding mechanisms of flow cytometry detection reagents is shown in **Figure 1A**. Firstly, since there was no limitation with the available sample volume, we evaluated the identification of our academic CD19 CAR-T cell product to determine optimal detection reagent (**Figure 1A**). We were able to compare four different reagents: CD19 CAR detection reagent from Miltenyi, CD19 protein and Protein L from Acrobiosystems and Anti-EGFR from Biolegend. T-cells from four healthy donors were transduced and academic CD19 CAR-T cell products were analyzed by flow cytometry. In **Figure 1B**, the percentage of detection is depicted for each reagent. For our academic CD19 CAR-T cell, the maximum percentage of detection is achieved with the anti-EGFR antibody with a similar detection level of the CD19 CAR detection reagent both for CD4+ and CD8+ cells (p=0.1142, p=0.6857). Besides, the detection with anti-EGFR allowed the highest resolution in CD4+ population (**Figure 1C**) (p=0.0283). In contrast, Protein L and CD19 protein from Acrobiosystems did not allow an optimal detection of our academic CD19 CAR-T cell (**Figure 1B**). Finally, for the evaluation of unspecific binding, untransduced T-cells (UTD) from the same donors were stained with the respective detection reagents to measure background staining in negative controls that presented no signal except for the CD8+ UTD stained with Protein L-FITC that showed some background signal (**Supplementary Figure 1**).

Once we validated the identification of our academic CD19 CAR-T cell, samples from three different patients infused with either Tisagenlecleucel or Axicabtagene ciloleucel were analyzed by flow cytometry using the commercially available CD19 detection reagents from Acrobiosystems and from Miltenyi. In **Figure 1D**, representative plot of one patient stained with both antibodies is shown. Leftover samples from randomly selected healthy donors who had no history of CAR-T cell treatment served as negative controls. Assay specificity was measured with no background staining observed in any of the healthy donors (**Supplementary Figure 2**). Similar results according to CAR+ percentage of detection were obtained with both reagents. However, the CAR detection reagent showed the highest resolution in contrast to CD19 protein both in CD4+ and CD8+ cells (stain index of 36.82 *vs.* 6.18, p=0.0286 and stain index of 16.49 *vs.* 3.42, p=0.0286, respectively) (**Figure 1E**). Accordingly, we validated CD19 CAR detection reagent as the optimal reagent for the following monitoring of commercial CAR-T cell expansion *in vivo*.

### 3.2 CAR-T cell kinetics and phenotype characterization

**Figure 2:**
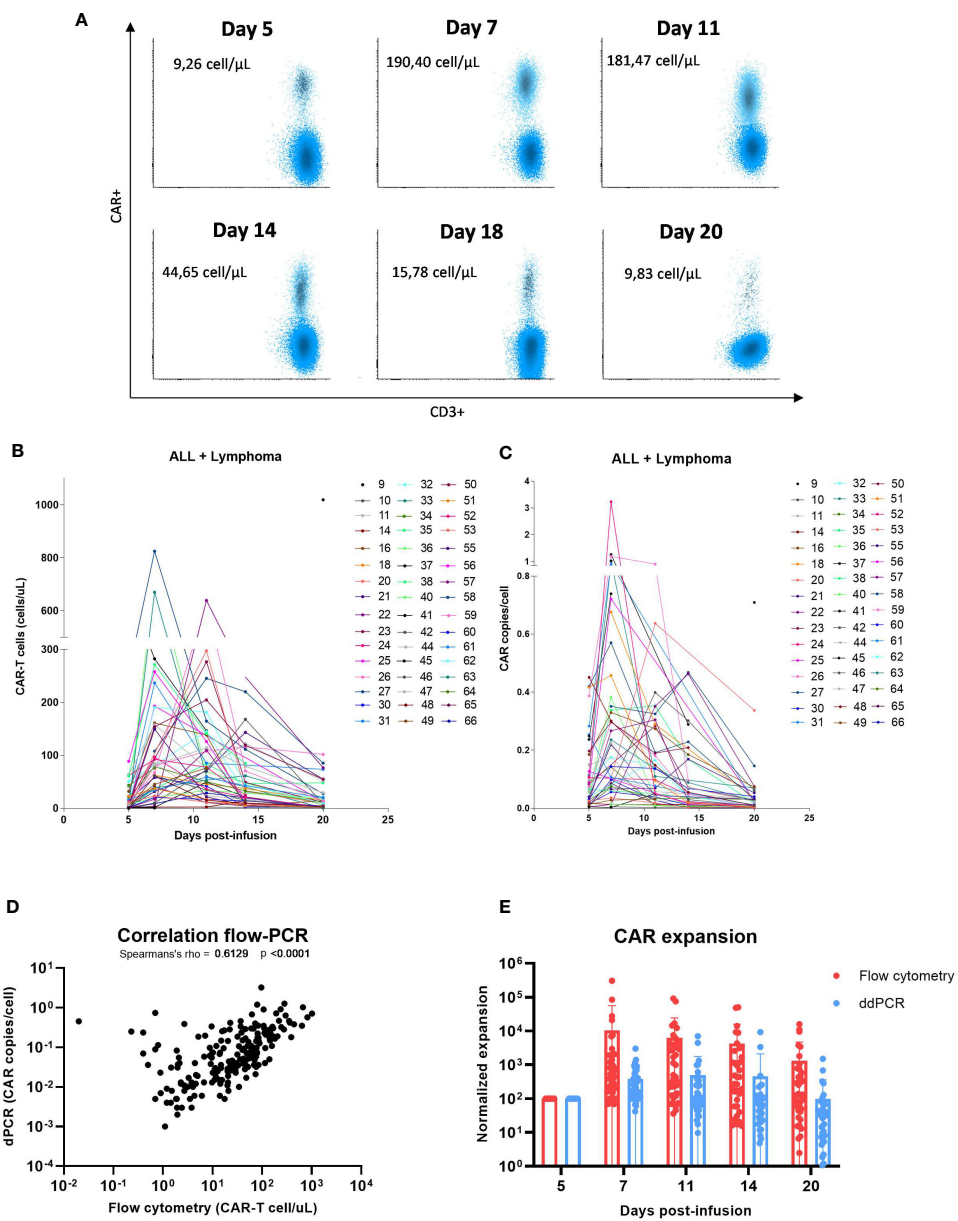
Commercial CD19 CAR-T cell expansion in the blood of patients post-infusion. **(A)** Flow cytometry plot of the expansion of CAR-T cells in the blood of patient 32 at different time points post-infusion. **(B)** Absolute count of CAR-T cells/uL by flow cytometry in the blood of patients with B-ALL and lymphoma. **(C)** CAR copies/cell measured by digital PCR in the blood of patients with B-ALL and lymphoma. **(D)** Spearman correlation in peripheral CAR-T cell expansion between flow cytometry and dPCR assays (n=48 patients and 185 observations). **(E)** Normalized peripheral CAR-T cell expansion by flow cytometry and dPCR assays (n=48 patients). Correlation was calculated using non-parametric Spearmans rank correlation coefficient.

Monitoring of peripheral blood commercial CAR-T cells and non-modified T cells post-infusion was performed in forty-eight patients with lymphoma and leukemia. The characteristics of patients included in this study are described in **Table 1**. The overall response rate (ORR) at 1-month post-infusion was 68.8% (**Table 1**). In **Figure 2A**, representative flow cytometry of the expansion of CAR-T cells in blood from patient 32 at different time points post-infusion is illustrated. Cellular kinetics of all patients show the same pattern of expansion with an exponential increase until days 7-11 followed by a rapid loss of CAR-T cell absolute count until day 20 post-infusion. The median day of peak expansion of CAR-T cells was day +7 (range of 5-14 days) after infusion, reaching a median of 112.2 CAR+ cells/uL (range of 0.7-1019 CAR+ cells/uL) in blood (**Figure 2B**). CAR-T cell expansion of patients with lymphoma, this is, excluding patients with LLA, is also shown (**Supplementary Figure 3A**). The median percentage of CAR+ cells within the CD3+ compartment in the peak expansion was 23.9% (n=48, range 0.3 to 82) (**Supplementary Figure 3C**). CAR-T cell expansion was also monitored by digital PCR. Similarly to flow cytometry data, kinetics of all patients shows the same pattern of expansion with an exponential increase until days 7-11 followed by a rapid loss of CAR-T cell absolute count until day 20 post-infusion. The median day of peak expansion of CAR-T cells was also day +7 (range of 5-14 days) after infusion, reaching a median of 0.245 CAR copies/cell (range of 0.03-3.23 CAR copies/cell) in blood (**Figure 2C** and **Supplementary Figure 3B**). Significant correlation was observed between flow cytometry and dPCR monitoring of CAR-T cells in blood (Spearman’s rho=0.61, p<0.0001) (**Figure 2D**). CAR-T cell expansion by flow cytometry and dPCR was compared at the different time points post-infusion with similar kinetics observed (**Figure 2E**). The expansion dynamics by flow cytometry and dPCR of patients infused with either Tisagenlecleucel (Tisa-cel) or Axicabtagene ciloleucel (Axi-cel) is depicted in **Supplementary Figure 4**. No significant differences were obtained by flow cytometry between both products in median peak expansion (p=0.4684) or median day of expansion (p=0.33) (**Supplementary Figure 4E**). However, significant increased CAR copies/cell at peak expansion were observed in patients infused with Axi-cel (p=0.0006) (**Supplementary Figure 4F**).

**Table 1:** Characteristics of the patients included in the study.

Patients (n)	48
PMBCL	8
DLBCL	26
FL	1
tFL	3
B-ALL	10
Median age (range)	51.5 (5-73)
Sex (%)	
Female	20 (41.7)
Male	28 (58.3)
Product (%)	
Tisagenlecleucel	18 (37.5)
Axicabtagene-ciloleucel	30 (62.5)
Previous lines (range)	3 (2-6)
Bridging therapy (%)	
YES	42 (87.5)
RT	2
CT	35
Inotuzumab	4
Steroids	1
NO	6 (12.5)
CRS grade (%)	97.9
0	1
1	24
2	21
3	2
ICANs grade (%)	45.8
0	26
1	12
2	4
3	4
4	2
Response D30 (%)	68.8
CR	19
PR	14
PD	9
SD	5

PMBCL (primary mediastinal B-cell lymphoma), DLBCL (diffuse large B-cell lymphoma), FL (follicular lymphoma), tFL (transformed follicular lymphoma), B-ALL (B-cell acute lymphoblastic leukemia), RT (radiotherapy), CT (chemotherapy), CRS (cytokine release syndrome), ICANs (immune effector cell-associated neurotoxicity syndrome), CR (complete response), PR (partial response), PD (progressive disease), SD (stable disease).

**Figure 3:**
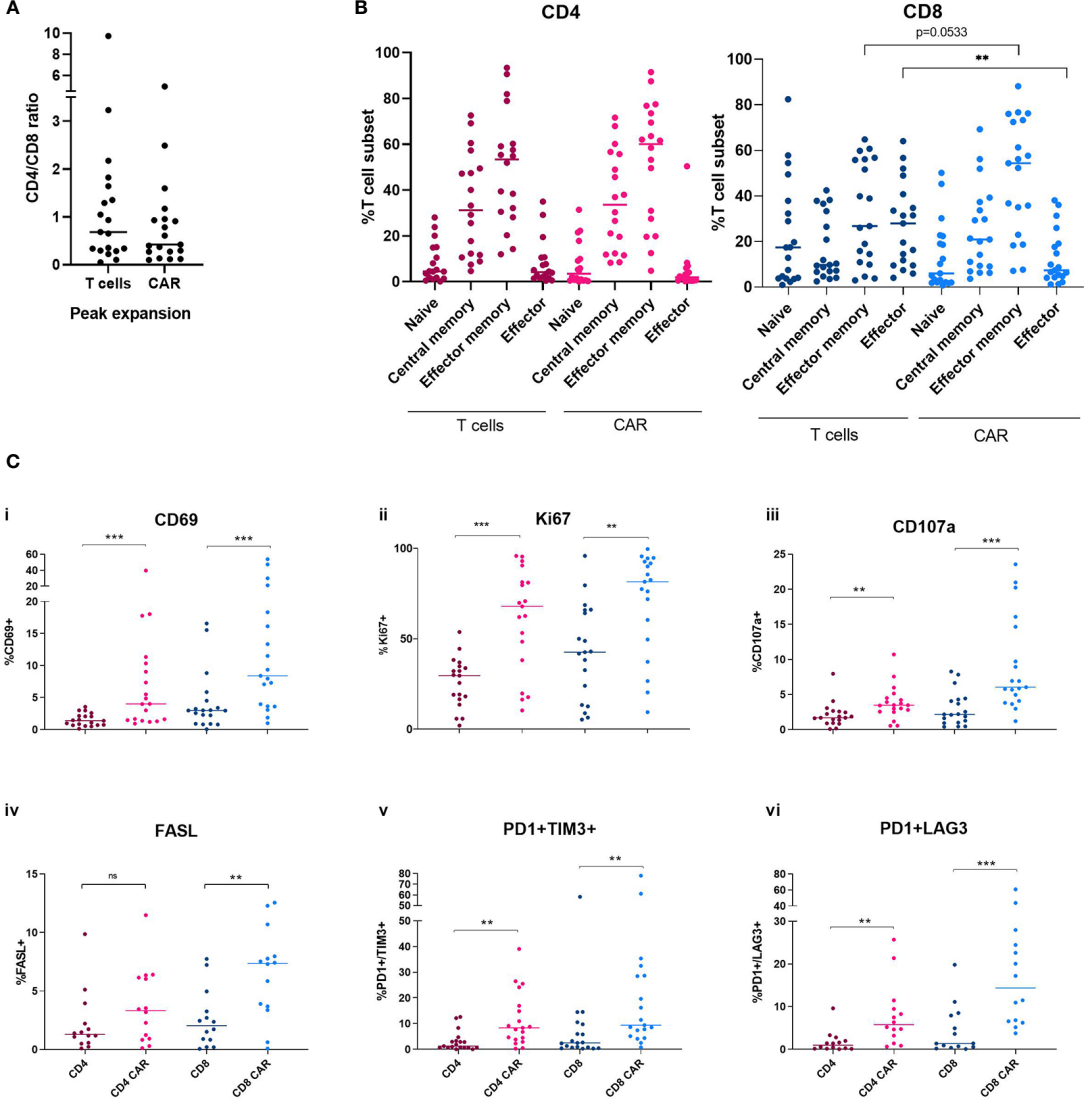
Immunophenotype characterization of non-modified T cells and CAR-T cells at the time of peak expansion in blood. **(A)** CD4/CD8 ratio of non-modified T cells and CAR-T cells at the time of peak expansion. **(B)** Memory subsets of CD4+ and CD8+ compartments comparing non-modified and CAR-T cells. **(C)** Levels of CD69 (i), Ki67 (ii), CD107a (iii), FasL (iv), PD1 and TIM3 (v) and PD1 and LAG3 (vi) in the CD4 and CD8 non-modified and CAR-T cells at the time of peak expansion. Depicted are median and individual values of the nine-teen samples. P-values between the indicated groups were calculated using unpaired Mann-Whitney U-t tests. ns: non-significant, **p<0.01, ***p<0.001.

Immunophenotyping of CAR+ cells and non-modified circulating T cells was performed at peak of expansion in each patient. The CD4/CD8 ratio was analyzed both in the CAR+ population and the circulating T-cells at the day of peak expansion. An increase in CD8+ population can be observed in the CAR+ population with a median CD4/CD8 ratio of 0.5 (**Figure 3A**). T cell subpopulations were identified as follows: naïve (CD62L+/CD45RO-/CD45RA+), central memory (CD62L+/CD45RO+/CD45RA), peripheral memory (CD62L-/CD45RO+/CD45RA-), effector (CD62L-/CD45RO-/CD45RA+). No significant differences were observed between CAR+ and non-modified circulating T-cells at the time of expansion in the CD4+ compartment. Among the CD8+ T cells, there was a decrease of effector cells in the CAR+ compared to the non-modified circulating T-cells accompanied by a trend towards an increase in memory phenotype in CAR+ cells (p=0.0056 and p=0.0533, respectively) (**Figure 3B**). Besides, CAR+ cells showed a significant increase in the expression of the activation marker CD69 compared to non-modified circulating T-cells both in CD4+ and CD8+ cells (p=0.0004, p=0.0008) (**Figure 3Ci**). The proliferation marker Ki67 and the cytotoxic marker CD107a were also significantly enhanced in CAR+ cells at the time of expansion in both CD4+ and CD8+ cells (p<0.01) (**Figures 3Cii, iii**). Regarding the exhaustion markers, CAR+ cells showed significant increased expression of the markers FasL, PD1+/TIM3+ and PD1+/LAG3+ compared to non-modified circulating T-cells both in CD4+ and CD8+ cells (p<0.05) (**Figures 3Civ, v, vi**). All these analyses were also performed excluding patients with B-ALL and we confirmed the same results (**Supplementary Figure 5**). All together, these data suggest a more activated, proliferative, cytotoxic, and exhausted phenotype of CAR+ cells at the time of peak expansion compared to non-modified circulating T-cells.

Immunophenotyping of CAR+ cells and non-modified circulating T cells was also compared between patients receiving Tisa-cel or Axi-cel products. Median CD4/CD8 ratio of CAR+ cells showed no significant differences between the two different products (median 0.4121 *vs*. 0.6100, respectively, p=0.7802). However, non-modified T cells from patients receiving Tisa-cel showed a tendency to an increased median CD4/CD8 ratio compared to patients receiving Axi-cel (median 0.9903 *vs*. 0.3375, respectively, p=0.1333) (**Supplementary Figure 6A**). T cell subsets were also analyzed considering both products. Non-modified T cells from patients infused with Tisa-cel showed a significantly increased proportion of effector CD4+ cells (p=0.0343). Besides, CD8+CAR+ cells from patients receiving Axi-cel showed a trend towards a reduced proportion of a central memory phenotype as compared to patients infused with Tisa-cel (p=0.0789) (**Supplementary Figure 6B**). Regarding the activation status, no differences were observed in the expression of CD69 between patients receiving Tisa-cel or Axi-cel products either in CAR+ or non-modified T cells CD4+ or CD8+ (**Supplementary Figure 6Ci**). A trend towards an increased Ki67 expression was observed in CAR+ cells of patients infused with Axi-cel products both in CD4+ and CD8+ compartments (p=0.0789) (**Supplementary Figure 6Cii**). No significant differences were observed between the different products regarding CD107a expression (**Supplementary Figure 6Ciii**). As far as the expression of co-inhibitory markers is concerned, we observed a trend towards an increased expression of FASL in CAR+ and non-modified T cells of patients infused with Axi-cel compared to Tisa-cel products both in CD4+ and CD8+ compartments (p=0.0263, p=0.1895, p=0.1128 and p=0.0653, respectively) (**Supplementary Figure 6Civ**). In line with this, a significantly increased expression of PD1+TIM3+ and PD1+LAG3+ was observed in CAR+ cells of patients infused with Axi-cel in both CD4+ and CD8+ cells (p=0.0279, p=0.0010, p=0.0220 and p=0.0789, respectively) (**Supplementary Figures 6Cv**, **6Cvi**). In summary, CAR+ cells from patients infused with Axi-cel products showed an increased CD4+ effector non-modified T cells, reduced CAR+CD8+ central memory, and increased proliferation and exhaustion at the time of peak expansion compared to patients receiving Tisa-cel products.

CD4/CD8 ratio was also compared between leukapheresis samples and non-modified T or CAR-T cells at the time of peak expansion. A tendency to a progressive reduction in CD4/CD8 ratio was observed from leukapheresis to CAR-T cells at the time of peak expansion (median 0.95 *vs.* 0.42) (**Supplementary Figure 7A**). T cell memory subsets were analyzed in CD4+ and CD8+ compartments comparing leukapheresis samples with non-modified T and CAR-T cells at peak expansion. A significant increase in effector memory and a reduction in naïve cells was observed both in CD4+ non-modified and CAR-T cells (p=0.0005, p=0.0047, p=0.0004, p=0.0013, respectively) (**Supplementary Figure 7B**). A tendency to reduced proportion in central memory cells was also observed in both non-modified T and CAR-T cells compared to leukapheresis in CD4+ compartment (p=0.08 and p=0.07, respectively) (**Supplementary Figure 7B**). CD8+ CAR-T cells showed a significant reduction in naïve and effector cells and an increase in effector memory cells compared to leukapheresis (p=0.05, p=0.0446 and p=0.0046, respectively) (**Supplementary Figure 7B**).

TCR repertoire was also evaluated in 10 patients to study clonality of CAR+ cells and non-transduced circulating T-cells (**Supplementary Figure 8**). Of these 10 patients, three of them relapsed (patients 30, 33 and 35). Focusing on patient 30, relapse occurred one month after CAR-T cell infusion. In this patient, the clones with the higher frequency in CAR+ cells compartment were Vb7.1, Vb17 and Vb21.3. In non-transduced circulating T-cells compartment, the clones observed with a higher frequency were Vb3, Vb13.6 and Vb8. A higher number of patients and longer follow up are required to evaluate the impact of TCR clonality after CAR-T cell infusion.

### 3.3 Correlation of CAR-T cell expansion *in vivo* with toxicity and event-free survival

**Figure 4:**
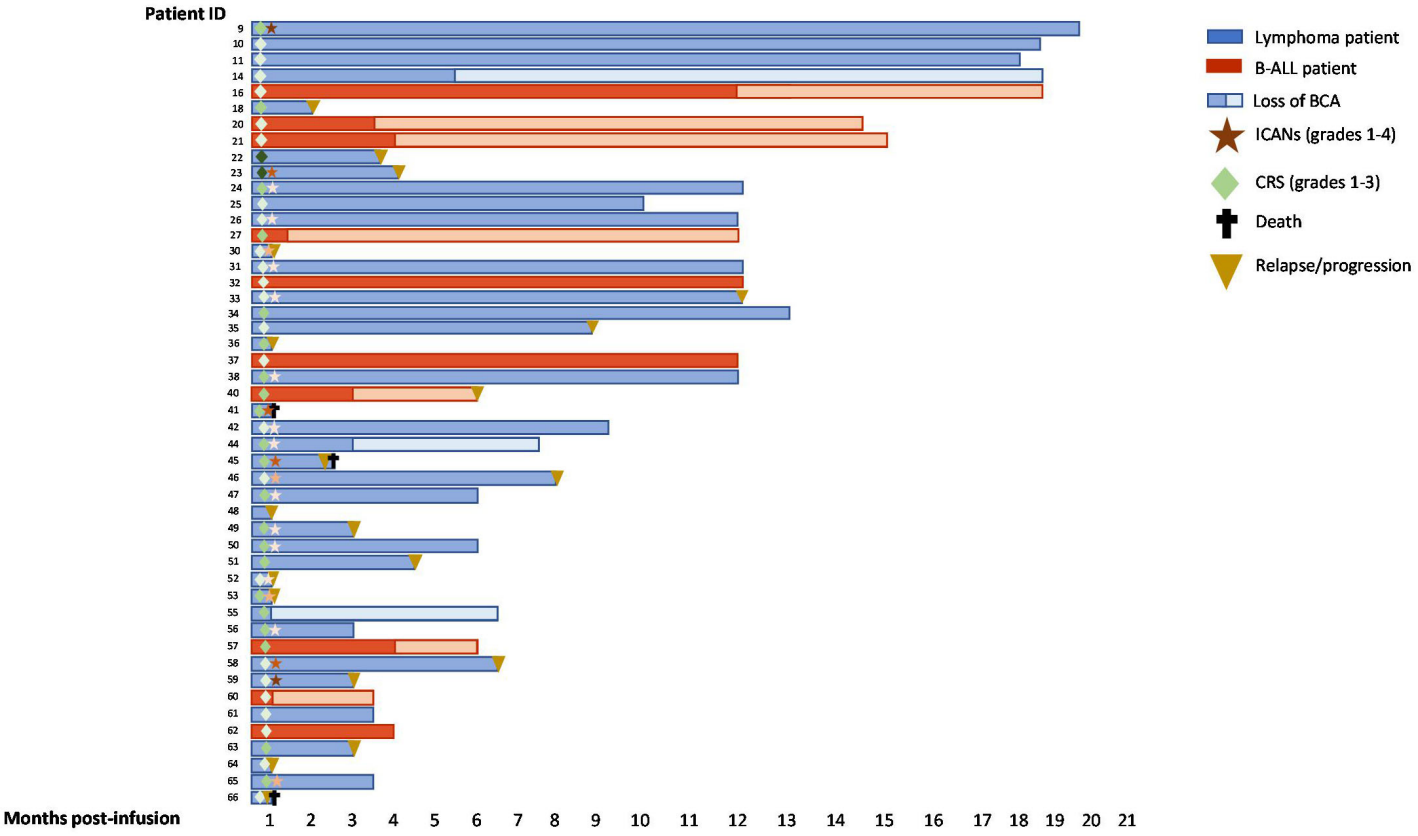
Swimmer plots of 48 patients with B-ALL or lymphoma after CD19 CAR-T cell infusion. BCA (B-cell aplasia). CRS and ICANs grades are depicted from 1-4 according to the intensity of the color.

Summary outcomes of each patient during the months post-infusion is depicted in **Figure 4** including toxicity, follow up, B-cell aplasia, relapse or death.

**Figure 5:**
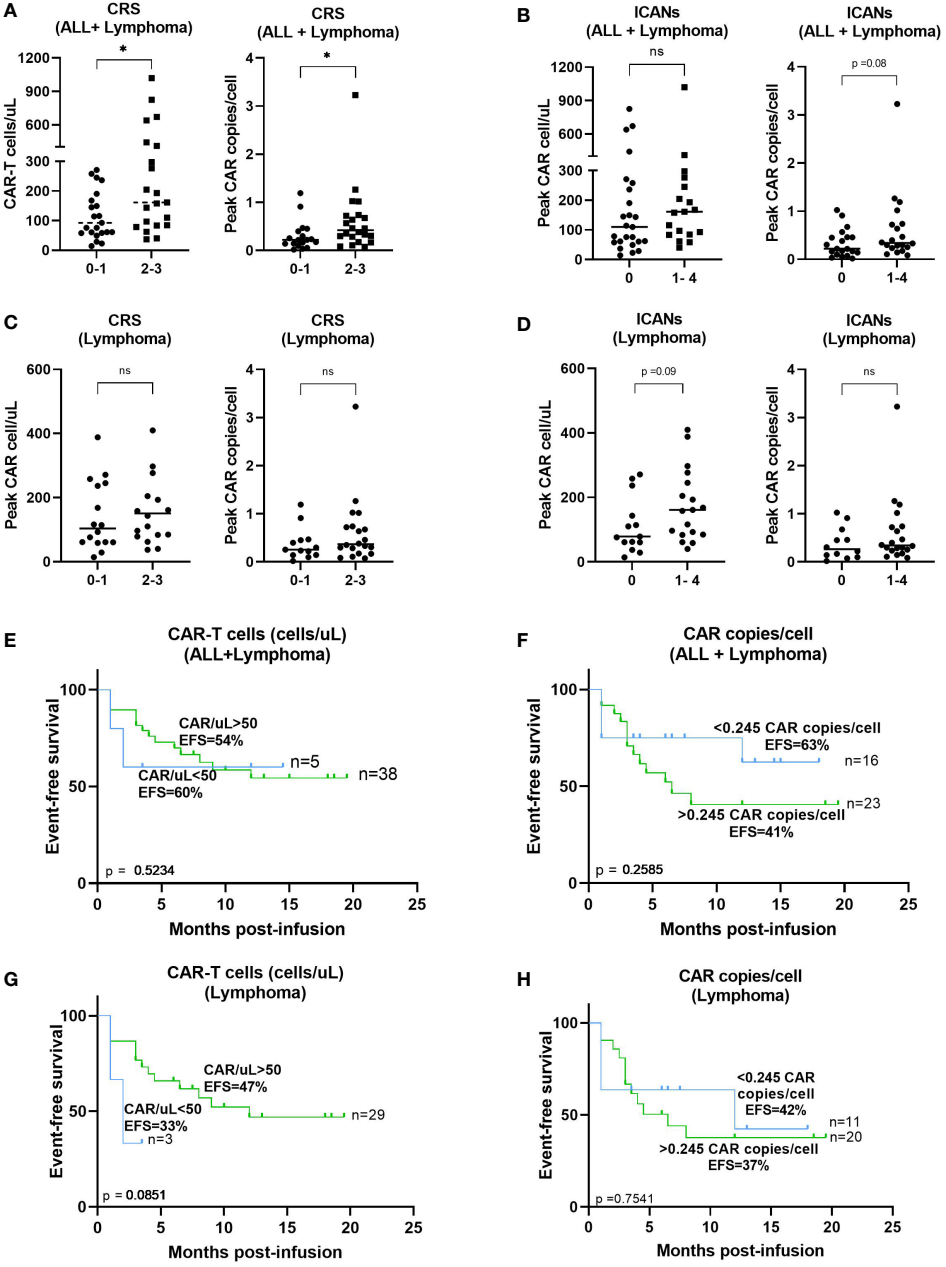
Correlation between CAR-T cell expansion in blood and toxicity or response. **(A)** Correlation between peak expansion by flow cytometry (*left)* or dPCR *(right)* and severity of CRS from patients with B-ALL and lymphoma. **(B)** Correlation between peak expansion by flow cytometry *(left)* or dPCR *(right)* and incidence of ICANs from patients with B-ALL and lymphoma. **(C)** Correlation between peak expansion by flow cytometry (*left)* or dPCR *(right)* and severity of CRS from patients with lymphoma excluding patients with B-ALL. **(D)** Correlation between peak expansion by flow cytometry *(left)* or dPCR *(right)* and incidence of ICANs from patients and lymphoma excluding patients with B-ALL. Correlation between absolute CAR-T cell count **(E)** or CAR copies/cell **(F)** and event free survival of all patients or **(F)** excluding patients with B-ALL **(G, H)**. Depicted are median and individual values of the forty-eight samples. P-values between the indicated groups were calculated using unpaired Mann-Whitney U-t tests. ns: non-significant, *p<0.05. Correlation studies were performed using Kaplan-Meier survival analysis.

Median peak expansion of patients was correlated with the grade of CRS or ICANs. Patients who developed grades 2-3 CRS showed a significantly increased peak of CAR-T cell absolute count *in vivo* (p=0.0261) as compared to those who did not develop it (**Figure 5A**). In addition, significant increased CAR copies/cell at the peak expansion were observed in patients with CRS grade 2-3 compared to patients with less severe CRS (p=0.0224) (**Figure 5A**). No significant differences were observed regarding the area under the curve (AUC) by flow cytometry between patients with CRS grades 2-3 compared to those with grades 0-1 (p=0.2758) but increased AUC by dPCR was observed in patients with grades 2-3 CRS (p=0.0299) (**Supplementary Figure 8A**). Besides, correlation between CAR-T cell expansion by flow cytometry and the appearance of ICANs was also performed but no significant differences were observed in median peak expansion (p=0.1787) (**Figure 5B**) or AUC (p=0.4212) (**Supplementary Figure 8B**). However, a trend towards an increased peak CAR copies/cell (p=0.08) and a significantly higher AUC by dPCR (p=0.0224) were observed in patients with some grade of ICANs (**Figure 5B** and **Supplementary Figure 9B**). Same analyses were performed including only those patients with lymphoma, but no significant differences were observed (**Figures 5C, D**, and **Supplementary Figures 9C, D**), although a trend towards an increased expansion in patients suffering from ICANs was observed (p=0.09). Correlation studies between CAR-T cell expansion *in vivo* and efficacy of the therapy were performed. However, no significant impact of CAR-T cells/uL on event-free survival was observed, neither for the peak expansion (p=0.5579, **Figure 5E**) or the area under the curve (p=0.2236, **Supplementary Figure 9E**). No significant correlation was observed between event-free survival and CAR copies/cell at the time of peak expansion or AUC (**Figure 5F** and **Supplementary Figure 9F**). Same analyses were performed including only those patients with lymphoma, but no significant differences were observed (**Figures 5G, H** and **Supplementary Figures 9G, H**).

Toxicity and efficacy of Tisa-cel and Axi-cel products were also compared. No significant differences were observed in the incidence of grades 2-3 CRS comparing patients infused with Tisa-cel (38.89%) or Axi-cel (53.33%) (p=0.3831) (**Supplementary Figure 10A**). However, an increased incidence of ICANs was observed in patients infused with Axi-cel (63.33%) as compared to patients receiving Tisa-cel (16.67%) (p=0.0025) (**Supplementary Figure 10B**). As far as the efficacy is concerned, no significant differences were observed in event-free survival comparing patients infused with Tisa-cel (EFS=63%) or Axi-cel (EFS=44%) (p=0.2539) (**Supplementary Figure 10C**).

### 3.4 Increased exhaustion markers at peak expansion correlate with increased event-free survival

**Figure 6:**
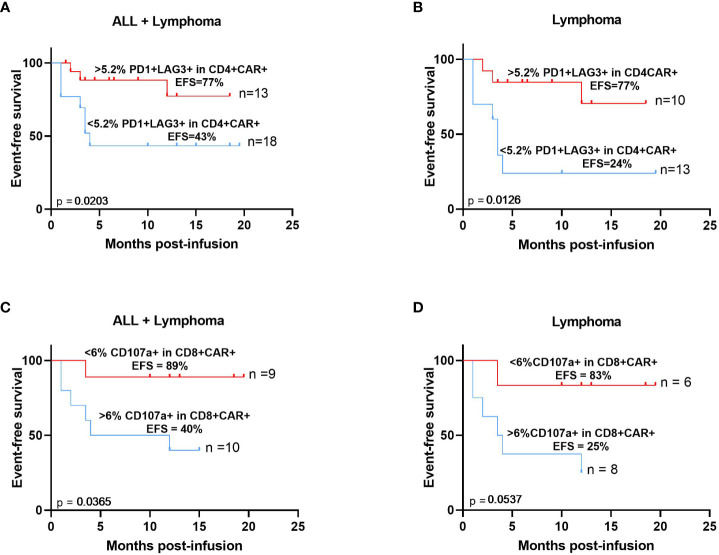
Immunophenotypic characteristics of CAR-T cells at the time of peak expansion correlate with increased event free-survival. Expression of PD1 and LAG3 >5.2% *(red)* within CD4 CAR-T cells at the time of peak expansion correlates with increased EFS in patients with B-ALL and lymphoma **(A)** or excluding patients with B-ALL **(B)**. Expression of CD107a <6% *(red)* within CD8+ CAR-T cells at the time of peak expansion correlates with increased EFS in patients with B-ALL and lymphoma **(C)** or excluding patients with B-ALL **(D)**. The median expression levels of these biomarkers were used to calculate these thresholds. Correlation studies were performed using Kaplan-Meier survival analysis.

Characterization of the immunophenotype of both CAR+ cells and non-transduced circulating T-lymphocytes was performed at day of peak expansion in nineteen patients with lymphoma and acute lymphoblastic leukemia. We found that patients with increased levels of PD1+LAG3+ cells (>5.2%) among the CD4+ CAR-T cell compartment correlated with an increased event-free survival (p=0.0203) (**Figure 6A**). Upon excluding those patients with acute lymphoblastic leukemia (n=8), the same result was confirmed with a significantly increased event-free survival of those patients with a higher exhaustion phenotype at the peak expansion (p=0.0126) (**Figure 6B**). Besides, reduced levels (<6%) of the cytotoxic marker CD107a+ among the CD8+CAR+ population was also associated with an increased event-free survival (p=0.0365) (**Figure 6C**). We also confirmed this result after excluding those patients with acute lymphoblastic leukemia (p=0.0537) (**Figure 6D**).

Together these data show how the monitoring and characterization of the phenotype of CAR-T cells *in vivo* may contribute to the identification of patients with worse prognosis very early after CAR-T cell infusion. Our results reveal that an increased exhaustion phenotype in the CAR-T cells at the peak expansion *in vivo* correlates with an increased event-free survival in patients with both lymphoma and leukemia.

## 4 Discussion

The correct detection, monitoring and characterization of CAR-T cells *in vivo* might be a fundamental tool for the identification of biomarkers associated with response or toxicity at very early time-points post-infusion which would serve to provide earlier interventions. In line with this, blood samples from 48 patients treated with CD19 CAR-T cells were analyzed by flow cytometry and digital PCR to count CAR+ cells *in vivo* and characterize their immunophenotype.

Validation of the detection method for the identification of CAR-T cells by flow cytometry is essential. Therefore, several commercially available reagents were tested for the identification of both academic and commercial CD19 CAR-T cells. These methods can be directed against the CAR itself or to gene markers incorporated into the construct. Besides, flow cytometry staining can be direct or indirect using secondary detection reagents. According to this, there are advantages and disadvantages that need to be considered to choose the optimal detection reagent. Our academic CD19 CAR-T cells incorporate the transduction marker EGFRt and we correctly identified these cells with both anti-EGFR and CD19 CAR detection reagent. However, neither CD19 protein nor protein L were able to properly detect our academic CAR-T cells. On the other hand, commercial CD19 CAR-T cells were correctly identified with both CD19 CAR detection reagent and CD19 protein. Of note, although our academic CD19 CAR contains the heavy variable (VH) and light variable (VL) chain of anti-CD19 monoclonal antibody FMC63, a codon optimization was performed. Moreover, different linker, hinge and transmembrane domains were used compared to commercial CD19 CARs (19, 20); therefore, these specific characteristics of our academic CD19 CAR could explain the observed differences in the detection with CD19 protein reagent. In both academic and commercial CAR-T cells, CD19 CAR detection reagent showed optimal results for the detection of these cells. This reagent contains a recombinantly expressed fusion protein consisting of the human CD19 extracellular domain and an optimized mutated human IgG1 Fc region. It employs indirect staining with a secondary step with Streptavidin which makes the flow cytometry protocol longer in time but also more adaptable to a complex multiplex staining panel. In summary, in our hands, the CD19 CAR detection reagent was the optimal identification method according to percentage of detection, stain index and specificity and we selected this reagent for the *in vivo* monitoring study. Also, with this study we point out the importance of the validation of the detection method for the identification of different CAR-T cell products by flow cytometry depending on their specific structure, even when these are directed against the same tumor antigen.

CD19 CAR-T cells were monitored in the blood of patients with lymphoma and leukemia infused with either Tisa-cel or Axi-cel. Although these two products present different co-stimulatory domains in their structure, Tisa-cel incorporates 4-1BB while Axi-cel contains CD28 as co-stimulatory domain; most of the patients showed a similar expansion pattern to that previously reported in other studies (3, 6, 7, 19, 20) with an exponential phase until days 7-11 post-infusion, followed by a rapid loss until day 20 post-infusion. In our study, similar expansion was obtained by flow cytometry with both commercial products with no significant differences in median peak CAR+ cell/uL or day of expansion, although, it seems to be a tendency to an earlier expansion in patients infused with Tisagenlecleucel (median day of expansion 7) compared to Axicabtagene ciloleucel (median day of expansion 9) (**Supplementary Figure 3**). Considering that pediatric patients with ALL can only be treated with Tisa-cel, we performed the same analysis excluding ALL patients and the median day of expansion remained at 7 days post-infusion for those adult patients infused with Tisa-cel (data not shown). Several preclinical studies have demonstrated functional differences between these two co-stimulatory domains that may also lead to differences *in vivo* (21–23). However, no clinical differences in efficacy or toxicity have been demonstrated between different co-stimulatory domains (24). Moreover, this difference in median day of peak expansion between the two commercial products was not significant as it has been described in other studies (25). We also monitored CAR-T cells in the same patients by digital PCR at the same time points. The median day of peak expansion of CAR-T cells was day +7 (range of 5-14 days) after infusion, reaching a median of 0.245 CAR copy numbers/cell (range of 0.03-3.23 CAR copies/cell) in blood. No significant differences were observed between Tisa-cel (median day of expansion d+7) compared to Axi-cel (median day of expansion d+7). Although CAR-T cell absolute count by flow cytometry was not significantly different between the two products, CAR copies/cell were significantly higher at the peak expansion in patients infused with Axi-cel. As other studies have previously showed, a significant correlation was observed between flow cytometry and dPCR assays (8, 26–28). Several studies have demonstrated that digital PCR shows a higher sensitivity compared to flow cytometry (17, 29) In the last years, optimization and standardization of the flow cytometry protocols with bulk lysis have increased flow cytometry sensitivity to 10^-5^ approaching to digital PCR (10^-7^). Besides, flow cytometry allows not only the detection and quantification of CAR-T cells post-infusion, but also the immunophenotypic characterization of these cells and the immune system of the patient at the same time. On the other hand, flow cytometry needs to be performed with fresh blood samples and requires qualified personnel for the analysis of the data.

At the time of peak expansion, phenotypic characterization was performed comparing CAR+ and non-modified circulating T-cells. CD4/CD8 ratio showed a reduction in CAR-T cells compared to non-modified circulating T-cells although it did not reach statistical significance. This data shows a predominant expansion of CD8+ versus CD4+ in CAR-T cells after infusion, independent of the CD4/CD8 ratio of the infused product, as it has been described in other studies (30, 31). The analysis of T-cell memory subsets showed significant differences between CAR-T and non-modified circulating T-cells. Both CD4+ and CD8+ CAR-T cells show predominant effector memory phenotype at the time of peak expansion. CD8+ CAR-T cells show significantly reduced levels of effector cells and a significant increase in the effector memory subset. This difference in the differentiation state of CD8+ CAR-T cells may be due to antigen-driven differentiation as it has been also appreciated at the time of peak expansion in other studies (30). However, it is important to point out that these CAR-T cells were analyzed in peripheral blood and, considering cell trafficking to lymph nodes, different results could have been obtained if lymph node biopsies would have been analyzed (30). Both CD4+ and CD8+ CAR-T cells showed increased levels of activation, cytotoxicity, apoptosis, and exhaustion (defined as the co-expression of one or more co-inhibitory receptors in the cell surface) compared to non-modified circulating T-cells at the time of peak expansion. This result agrees with other studies in which CAR-T cells present a more differentiated phenotype compared to non-modified circulating T-cells at the time of peak expansion (30, 32, 33). At this time after infusion, CAR-T cells may have an elevated activity after rapid expansion and elimination of tumor cells and may have therefore acquired a more activated, cytotoxic, and exhausted phenotype. Altogether these data indicate that, at the time of peak expansion, CAR-T cells have been through an antigen-driven differentiation leading to a reduced CD4/CD8 ratio, an increased effector memory phenotype and the acquisition of an activated and exhausted phenotype (30, 34–36). It is important to remark that, although there are several studies about the phenotypic characterization of CAR-T cell in the leukapheresis and infusion products (6, 13, 25, 37), scarce information is available about the phenotypic characterization of CAR-T cells at the time of peak expansion as the current study shows.

Different studies have shown clinical differences between Tisa-cel and Axicel; nevertheless, scarce information is available regarding the differences in the immunophenotype of CAR-T cells comparing these two products and most studies available are focused on the evaluation of the infusion products (38). With this background, in the current study immunophenotypic characterization of non-modified T cells and CAR-T cells was compared between both products at the time of peak expansion. Patients receiving Axi-cel products showed both increased proliferation and increased expression of co-inhibitory receptors at the time of peak expansion in non-modified T and CAR-T cells from both CD4+ and CD8+ compartments. These data suggest that antigen-driven differentiation process may be augmented in patients infused with Axi-cel compared to Tisa-cel products.

Analysis of the leukapheresis samples as compared to circulating non-modified T or CAR-T cells showed a reduction in CD4/CD8 ratio in CAR-T cells at the time of peak expansion. Besides, in both CD4+ and CD8+ compartments a reduction in naïve and an increase in effector memory cells was observed. Furthermore, a tendency to reduced central memory was also observed in both CD4+ and CD8+ compartments at the time of peak expansion. Altogether these data may indicate that, compared to the leukapheresis, both non-modified T cells and CAR-T cells display a more differentiated T cell phenotype, which could be attributed to stimulation post-antigen exposure. These data agree with previous studies showing that CAR-T cells at the time of peak expansion show an inversed CD4/CD8 ratio and a low proportion of naïve and central memory compared to healthy donors and peripheral blood samples of patients before CAR-T cell infusion (27, 39)

Cell kinetics monitoring has been performed in multiple studies providing inconsistent results about the correlation with response or toxicity. Although more agreement exists about the correlation of CAR-T cell expansion with increased tumor burden (31, 40, 41), it is not that clear whether there is a correlation between CAR-T expansion and efficacy, toxicity, or CAR-T cell dose. Some studies have demonstrated that *in vivo* CAR-T cell expansion correlates with higher grades of ICANs and CRS (1, 8, 31, 41–43). In our study, correlation between peak CAR-T in blood and more severe CRS was observed in agreement with previous studies. However, no correlation was observed between AUC and CRS severity. Although no significant correlation was observed with neurotoxicity, a trend towards an increased median peak expansion of CAR-T cell absolute count and CAR copies/cell was observed in patients suffering some grade of ICANs. Regarding the efficacy of the therapy, no correlation was observed between CAR-T peak expansion or AUC in blood and event-free survival. In line with this, there is controversy in relation to the association of *in vivo* CAR-T expansion and efficacy of CAR-T cell therapy. Numerous studies have performed CAR-T cell kinetics and inconsistent results have been shown with regards to response. Some studies have shown that patients achieving CR display significant increased CAR-T expansion in blood compared to NR patients (6, 25, 40, 41, 44, 45). However, there are other studies confirming no correlation between peak expansion and response to CAR-T cells (8–10). This variability may be due to different factors including clinical characteristics of patients, the CAR-T cell constructs considered, previous CAR-T therapies or the tumor antigen targeted by CAR-T cells (32).

The comparison between Tisa-cel versus Axi-cel in terms of toxicity or efficacy showed no differences in terms of severe CRS incidence but an increased incidence of ICANs in patients receiving Axi-cel products. This agrees with previous studies that have reported increased toxicity in patients infused with Axi-cel (37, 46, 47). However, no significant differences were observed in event-free survival of patients infused with Tisa-cel or Axi-cel products.

Immunophenotypic characterization of CAR-T cells has been analyzed in several studies but mainly at the time of leukapheresis or infusion products (6, 32, 48). Little information is available about the immunophenotype of CAR-T cells during *in vivo* expansion or at the time of peak expansion (30). In the same way, scarce information is available regarding the correlation of immunophenotype of CAR-T cells and the prognosis of patients at the long-term. In this study, two biomarkers were shown to correlate with increased event-free survival at the time of peak expansion, increased PD1+LAG3+ in CD4+ CAR-T cells and reduced CD107a+ in CD8+ CAR-T cells. Exhausted state has been commonly associated as unresponsive or nonfunctional state of T cells achieved during chronic virus infection. It is defined as the elevated and prolonged co-expression of many inhibitory receptors, such as PD1, CTLA4, TIM3, or LAG3 in the cell membrane after antigen exposure; specific epigenetic landscape and metabolism; and loss of effector function. T cell exhaustion occurs when infection continuous after effector phase and persistent and chronic antigen stimulation happens. Normal effector cells express several inhibitory receptors during late stages of normal activation to balance immune responses (49–51). Several studies have analyzed exhaustion markers in CAR-T cells at the time of leukapheresis and in the infusion products demonstrating that increased exhaustion markers at these time points correlate with reduce efficacy of CAR-T cells (6, 9, 12, 13, 33, 52, 53). However, little information is available about the exhaustion markers of CAR-T cells in the blood of patients post-infusion. Some studies have analyzed the memory populations or the levels of CAR-Treg (8, 30, 33). In this study we demonstrate a correlation between increased levels of PD1+LAG3+ in CD4+ CAR-T cells and increased event-free survival. We propose that CAR-T cells from these patients have increased levels of co-inhibitory receptors (immune checkpoint molecules) at peak expansion following antigen-driven activation that has allowed the elimination of tumor cells and though permits better disease control that correlates with increased event-free survival. Therefore, the timing in the analysis of these co-inhibitory receptors would be essential to understand the dynamics of CAR-T cell activation and its correlation with efficacy. In this way, increased levels of co-inhibitory receptors prior to antigen stimulation would associate with reduced efficacy but at the time of peak expansion, when CAR-T cells have already interacted with tumor cells and made their function, would correlate with increased response. According to our proposed model, co-inhibitory receptors would increase during *in vivo* expansion due to CAR-T cells correct functioning and would downmodulate after peak expansion in blood. In this case, exhaustion markers should also be analyzed at longer time points post-infusion to follow the expression dynamics and confirm downregulation after antigen clearance. At the same time, we have shown that patients with reduced levels of CD107a+ in CD8+ CAR-T cells at the time of peak expansion presented increased event-free survival. CD107a or lysosomal associated membrane protein 1 (LAMP-1) is a major component of the lysosomal membrane (54) and its expression in the T cell surface correlates with cytolytic effect (55). Upon target-recognition in T cells, pre-formed cytotoxic granules in the cytoplasm circulate to the site of cell-cell contact in the plasma membrane and secrete effector molecules, such as perforin and granzyme, into the cytotoxic immunological synapse to clear target cells. As degranulation occurs, secretory lysosomes are released and CD107a is expressed in the cell membrane accessible to antibody binding as evidence of cytolytic effect (55). Therefore, CD107a pattern of expression on the cell membrane is transient depending on antigen stimulation. After stimulation, CD107a levels rapidly increase reaching maximum at about 4 hours later and then decreasing due to internalization of the protein (56). Some studies have revealed CD107a may not only be a marker of degranulation, but it has also a role in the trafficking of lytic granules to the cell membrane (57). In summary, CD107a is a highly dynamic marker that has been shown to be transient in the cell membrane. Upon degranulation, CD107a is externalized to the cell membrane and cells become positive for cell surface CD107a for a brief period of time before this protein is rapidly retrieved by the endocytic pathway (56). According to this, we propose that during CAR-T cell expansion, *in vivo* levels of CD107a would increase at early time points post-infusion due to antigen-recognition and once the cytotoxic effect has been performed these levels would decrease in the cell membrane. Therefore, those patients with reduced levels of CD107a in CD8+ CAR-T cells at the time of peak expansion would be those with a higher activation and increased CAR-T cytotoxic effect at earlier time points, thus correlating with an increased event-free survival. In conclusion, immune cell studies, and correct interpretation of the dynamics of the immunophenotype during CAR-T cell therapy (from leukapheresis to manufacturing and post-infusion monitoring) is essential for the understanding of CAR-T cells performance *in vivo* and for the detection of biomarkers that would identify those patients with worse prognosis.

In summary, despite the great results of CAR-T cell therapy in the treatment of relapse/refractory lymphoma and LLA, only less than 50% of patients retain long-term response and many challenges need to be solved to increase this rate. This study stands out the importance of the *in vivo* monitoring and characterization of these CAR-T cells for the identification of patients with worse prognosis at very early time post-infusion and highlights the predictive value of phenotypic characteristics at the time of peak expansion. To the best of our knowledge, our study shows for the first time the correlation of increased levels of co-inhibitory receptors and reduced expression of cytotoxicity markers at the time of peak expansion in CAR-T cells, with an increased event-free survival in patients with lymphoma and acute lymphoblastic leukemia.

## Data availability statement

The original contributions presented in the study are included in the article/**Supplementary Material**. Further inquiries can be directed to the corresponding author.

## Ethics statement

The studies involving human participants were reviewed and approved by Virgen del Rocío University Hospital, Seville (Spain). Written informed consent to participate in this study was provided by the participants’ legal guardian/next of kin.

## Author contributions

CB-GC designed and performed experiments and analyzed data. B S-M designed and performed experiments, analyzed data and wrote the manuscript. EG-G contributed in the experiment design and critically reviewed the manuscript. LS-F contributed in the data analysis. RM-G, JG-L, JL-S and MG-E contributed in data collection and analysis. VR-M helped in the sample collection and data analysis. JD-S, AM-Q, J-RO and CB-G contributed in data collection. MRJ-L, BG-A and IC-B contributed in the data analysis. MR-S contributed in the data analysis. JB served as scientific advisor. JP-S and TC-V designed experiments and project and critically reviewed the manuscript.

## References

[1] Park JH Rivière I Gonen M Wang X Sénéchal B Curran KJ. Long-term follow-up of CD19 CAR therapy in acute lymphoblastic leukemia. New Engl J Med (2018) 378:449–59. doi: 10.1056/NEJMOA1709919/SUPPL_FILE/NEJMOA1709919_DISCLOSURES.PDF PMC6637939 29385376

[2] García-Guerrero E Sierro-Martínez B Pérez-Simón JA. Overcoming chimeric antigen receptor (CAR) modified T-cell therapy limitations in multiple myeloma. Front Immunol (2020) 11:1128. doi: 10.3389/fimmu.2020.01128 32582204 PMC7290012

[3] Mueller KT Maude SL Porter DL Frey N Wood P Han X Waldron E Chakraborty A Awasthi R Levine BL Melenhorst JJ Grupp SA June CH Lacey SF. Cellular kinetics of CTL019 in relapsed/refractory B-cell acute lymphoblastic leukemia and chronic lymphocytic leukemia. Blood (2017) 130 (21):2317–25 doi: 10.1182/blood-2017-06-786129 PMC5731220 28935694

[4] Lee DW Santomasso BD Locke FL Ghobadi A Turtle CJ Brudno JN. ASTCT consensus grading for cytokine release syndrome and neurologic toxicity associated with immune effector cells. Biol Blood Marrow Transplant (2019) 25:625–38. doi: 10.1016/J.BBMT.2018.12.758 PMC12180426 30592986

[5] Gust J Ponce R Liles WC Garden GA Turtle CJ. Cytokines in CAR T cell-associated neurotoxicity. Front Immunol (2020) 11:577027. doi: 10.3389/FIMMU.2020.577027 33391257 PMC7772425

[6] Fraietta JA Lacey SF Orlando EJ Pruteanu-Malinici I Gohil M Lundh S. Determinants of response and resistance to CD19 chimeric antigen receptor (CAR) T cell therapy of chronic lymphocytic leukemia. Nat Med (2018) 24:563–71. doi: 10.1038/s41591-018-0010-1 PMC6117613 29713085

[7] Mueller KT Waldron E Grupp SA Levine JE Laetsch TW Pulsipher MA. Clinical trials: Immunotherapy clinical pharmacology of tisagenlecleucel in b-cell acute lymphoblastic leukemia. Clin Cancer Res (2018) 24 (24):6175–84 doi: 10.1158/1078-0432.CCR-18-0758 PMC7433345 30190371

[8] Good Z Spiegel JY Sahaf B Malipatlolla MB Ehlinger ZJ Kurra S. Post-infusion CAR TReg cells identify patients resistant to CD19-CAR therapy. Nat Med (2022) 28:1860–71. doi: 10.1038/s41591-022-01960-7 PMC10917089 36097223

[9] Schuster SJ Bishop MR Tam CS Waller EK Borchmann P McGuirk JP. Tisagenlecleucel in adult relapsed or refractory diffuse Large b-cell lymphoma. New Engl J Med (2019) 380:45–56. doi: 10.1056/NEJMOA1804980/SUPPL_FILE/NEJMOA1804980_DISCLOSURES.PDF 30501490

[10] Sauter CS Senechal B Rivière I Ni A Bernal Y Wang X. CD19 CAR T cells following autologous transplantation in poor-risk relapsed and refractory b-cell non-Hodgkin lymphoma. Blood (2019) 134:626–35. doi: 10.1182/BLOOD.2018883421 PMC6695562 31262783

[11] Cohen AD Garfall AL Stadtmauer EA Melenhorst JJ Lacey SF Lancaster E. B cell maturation antigen–specific CAR T cells are clinically active in multiple myeloma. J Clin Invest (2019) 129:2210–21. doi: 10.1172/JCI126397 PMC6546468 30896447

[12] Deng Q Han G Puebla-Osorio N Ma MCJ Strati P Chasen B. Characteristics of anti-CD19 CAR T cell infusion products associated with efficacy and toxicity in patients with large b cell lymphomas. Nat Med (2020) 26:1878–87. doi: 10.1038/s41591-020-1061-7 PMC8446909 33020644

[13] Finney OC Brakke H Rawlings-Rhea S Hicks R Doolittle D Lopez M. CD19 CAR T cell product and disease attributes predict leukemia remission durability. J Clin Invest (2019) 129:2123–32. doi: 10.1172/JCI125423 PMC6486329 30860496

[14] Kunz A Gern U Schmitt A Neuber B Wang L Hückelhoven-Krauss A. Optimized assessment of qPCR-based vector copy numbers as a safety parameter for GMP-grade CAR T cells and monitoring of frequency in patients. Mol Ther Methods Clin Dev (2020) 17:448–54. doi: 10.1016/j.omtm.2020.02.003 PMC7078460 32201711

[15] Fehse B Badbaran A Berger C Sonntag T Riecken K Geffken M. Digital PCR assays for precise quantification of CD19-CAR-T cells after treatment with axicabtagene ciloleucel. Mol Ther Methods Clin Dev (2020) 16:172–8. doi: 10.1016/j.omtm.2019.12.018 PMC7005515 32055645

[16] Park C Lee J ul Hassan Z Ku KB Kim SJ Kim HG. Comparison of digital PCR and quantitative PCR with various SARS-CoV-2 primer-probe sets. J Microbiol Biotechnol (2021) 31:358–67. doi: 10.4014/JMB.2009.09006 PMC9705847 33397829

[17] Schanda N Sauer T Kunz A Hückelhoven-Krauss A Neuber B Wang L Hinkelbein M Sedloev D He B Schubert ML Müller-Tidow C Schmitt M Schmitt A. Sensitivity and specificity of CD19.CAR-T cell detection by flow cytometry and PCR. Cells (2021) 10 (11):3208. doi: 10.3390/cells10113208 PMC8621201 34831430

[18] Maryamchik E Gallagher KME Preffer FI Kadauke S Maus M v. New directions in chimeric antigen receptor T cell [CAR-T] therapy and related flow cytometry. Cytometry B Clin Cytom (2020) 98:299–327. doi: 10.1002/cyto.b.21880 32352629

[19] Hudecek M Lupo-Stanghellini MT Kosasih PL Sommermeyer D Jensen MC Rader C. Receptor affinity and extracellular domain modifications affect tumor recognition by ROR1-specific chimeric antigen receptor T cells. Clin Cancer Res (2013) 19:3153–64. doi: 10.1158/1078-0432.CCR-13-0330/85570/AM/RECEPTOR-AFFINITY-AND-EXTRACELLULAR-DOMAIN PMC3804130 23620405

[20] García-Guerrero E Rodríguez-Lobato LG Sierro-Martínez B Danhof S Bates S Frenz S. ATRA works synergistically with the γ-secretase inhibitor crenigacestat to augment BCMA on multiple myeloma and the efficacy of BCMA-CAR T-cells. Haematologica (2023) 108 (1):568–80 doi: 10.3324/haematol.2022.281339 PMC9890012 36722406

[21] Priceman SJ Gerdts EA Tilakawardane D Kennewick KT Murad JP Park AK. Co-Stimulatory signaling determines tumor antigen sensitivity and persistence of CAR T cells targeting PSCA+ metastatic prostate cancer. Oncoimmunology (2018) 7:e1380764. doi: 10.1080/2162402X.2017.1380764/SUPPL_FILE/KONI_A_1380764_SM7464.PDF PMC5749625 29308300

[22] Guedan S Posey AD Shaw C Wing A Da T Patel PR. Enhancing CAR T cell persistence through ICOS and 4-1BB costimulation. JCI Insight (2018) 3:e96976. doi: 10.1172/JCI.INSIGHT.96976 PMC5821198 29321369

[23] Milone MC Fish JD Carpenito C Carroll RG Binder GK Teachey D. Chimeric receptors containing CD137 signal transduction domains mediate enhanced survival of T cells and increased antileukemic efficacy. vivo. Mol Ther (2009) 17:1453–64. doi: 10.1038/MT.2009.83 PMC2805264 19384291

[24] Cappell KM Kochenderfer JN. A comparison of chimeric antigen receptors containing CD28 versus 4-1BB costimulatory domains. Nat Rev Clin Oncol (2021) 18:715–27. doi: 10.1038/S41571-021-00530-Z 34230645

[25] Monfrini C Federico S Aragona V Magni M Ljevar S Vella C. Phenotypic Composition of Commercial Anti-CD19 CAR T Cells Affects In Vivo Expansion and Disease Response in Patients with Large B-cell Lymphoma. Clin Cancer Res (2022) 28 (15):3378–86. doi: 10.1158/1078-0432.CCR-22-0164 PMC9662896 35583610

[26] Badbaran A Berger C Riecken K Kruchen A Geffken M Müller I. Accurate in-vivo quantification of CD19 CAR-T cells after treatment with axicabtagene ciloleucel (Axi-cel) and tisagenlecleucel (Tisa-cel) using digital PCR. Cancers 2020 Vol 12 Page 1970 (2020) 12:1970. doi: 10.3390/CANCERS12071970 PMC7409173 32698364

[27] Peinelt A Bremm M Kreyenberg H Cappel C Banisharif-Dehkordi J Erben S. Monitoring of circulating CAR T cells: Validation of a flow cytometric assay, cellular kinetics, and phenotype analysis following tisagenlecleucel. Front Immunol (2022) 13:830773. doi: 10.3389/fimmu.2022.830773 35309367 PMC8926389

[28] Haderbache R Warda W Hervouet E da Rocha MN Trad R Allain V. Droplet digital PCR allows vector copy number assessment and monitoring of experimental CAR T cells in murine xenograft models or approved CD19 CAR T cell-treated patients. J Transl Med (2021) 19:1–13. doi: 10.1186/S12967-021-02925-Z/FIGURES/5 34154602 PMC8215786

[29] Reichman A Kunz A Joedicke JJ Höpken UE Keib A Neuber B. Comparison of FACS and PCR for detection of BCMA-CAR-T cells. Int J Mol Sci (2022) 23:903. doi: 10.3390/ijms23020903 35055086 PMC8777942

[30] Talleur AC Qudeimat A Métais J-Y Langfitt D Mamcarz E Crawford JC. Preferential expansion of CD8+ CD19-CAR T cells postinfusion and the role of disease burden on outcome in pediatric b-ALL. Blood Adv (2022) 6:5737–49. doi: 10.1182/BLOODADVANCES.2021006293 PMC9647829 35446934

[31] Turtle CJ Hanafi LA Berger C Gooley TA Cherian S Hudecek M. CD19 CAR–T cells of defined CD4+:CD8+ composition in adult b cell ALL patients. J Clin Invest (2016) 126:2123–38. doi: 10.1172/JCI85309 PMC4887159 27111235

[32] Shah NN Johnson BD Schneider D Zhu F Szabo A Keever-Taylor CA. Bispecific anti-CD20, anti-CD19 CAR T cells for relapsed b cell malignancies: a phase 1 dose escalation and expansion trial. Nat Med 2020 26:10 (2020) 26:1569–75. doi: 10.1038/s41591-020-1081-3 33020647

[33] Haradhvala NJ Leick MB Maurer K Gohil SH Larson RC Yao N. Distinct cellular dynamics associated with response to CAR-T therapy for refractory b cell lymphoma. Nat Med 2022 28:9 (2022) 28:1848–59. doi: 10.1038/s41591-022-01959-0 PMC9509487 36097221

[34] Kaech SM Wherry EJ Ahmed R. Effector and memory T-cell differentiation: implications for vaccine development. Nat Rev Immunol 2002 2:4 (2002) 2:251–62. doi: 10.1038/nri778 12001996

[35] Sullivan BM Juedes A Szabo SJ von Herrath M Glimcher LH. Antigen-driven effector CD8 T cell function regulated by T-bet. Proc Natl Acad Sci U.S.A. (2003) 100:15818–23. doi: 10.1073/PNAS.2636938100 PMC307651 14673093

[36] Paixão EA Barros LRC Fassoni AC Almeida RC. Modeling patient-specific CAR-T cell dynamics: Multiphasic kinetics *via* phenotypic differentiation. Cancers (Basel) (2022) 14:5576. doi: 10.3390/CANCERS14225576 36428671 PMC9688514

[37] Bachy E le Gouill S di Blasi R Sesques P Manson G Cartron G. A real-world comparison of tisagenlecleucel and axicabtagene ciloleucel CAR T cells in relapsed or refractory diffuse large b cell lymphoma. Nat Med (2022) 28 (10):2145–54. doi: 10.1038/s41591-022-01969-y PMC9556323 36138152

[38] Monfrini C Stella F Aragona V Magni M Ljevar S Vella C. Phenotypic composition of commercial anti-CD19 CAR T cells affects *In vivo* expansion and disease response in patients with Large b-cell lymphoma. Clin Cancer Res (2022) 28:3378. doi: 10.1158/1078-0432.CCR-22-0164 35583610 PMC9662896

[39] Blache U Weiss R Boldt A Kapinsky M Blaudszun AR Quaiser A. Advanced flow cytometry assays for immune monitoring of CAR-T cell applications. Front Immunol (2021) 12:658314. doi: 10.3389/fimmu.2021.658314 34012442 PMC8127837

[40] Locke FL Rossi JM Neelapu SS Jacobson CA Miklos DB Ghobadi A. Tumor burden, inflammation, and product attributes determine outcomes of axicabtagene ciloleucel in large b-cell lymphoma. Blood Adv (2020) 4:4898–911. doi: 10.1182/BLOODADVANCES.2020002394 PMC7556133 33035333

[41] Lee DW Kochenderfer JN Stetler-Stevenson M Cui YK Delbrook C Feldman SA. T Cells expressing CD19 chimeric antigen receptors for acute lymphoblastic leukaemia in children and young adults: A phase 1 dose-escalation trial. Lancet (2015) 385:517–28. doi: 10.1016/S0140-6736(14)61403-3 PMC7065359 25319501

[42] Hay KA Hanafi LA Li D Gust J Liles WC Wurfel MM. Kinetics and biomarkers of severe cytokine release syndrome after CD19 chimeric antigen receptor-modified T-cell therapy. Blood (2017) 130:2295–306. doi: 10.1182/BLOOD-2017-06-793141 PMC5701525 28924019

[43] Teachey DT Lacey SF Shaw PA Melenhorst JJ Maude SL Frey N. Identification of predictive biomarkers for cytokine release syndrome after chimeric antigen receptor T-cell therapy for acute lymphoblastic leukemia. Cancer Discovery (2016) 6:664–79. doi: 10.1158/2159-8290.CD-16-0040/42478/AM/IDENTIFICATION-OF-PREDICTIVE-BIOMARKERS-FOR PMC5448406 27076371

[44] Locke FL Ghobadi A Jacobson CA Miklos DB Lekakis LJ Oluwole OO. Long-term safety and activity of axicabtagene ciloleucel in refractory large b-cell lymphoma (ZUMA-1): a single-arm, multicentre, phase 1–2 trial. Lancet Oncol (2019) 20:31–42. doi: 10.1016/S1470-2045(18)30864-7 30518502 PMC6733402

[45] Locke FL Miklos DB Jacobson CA Perales M-A Kersten M-J Oluwole OO. Axicabtagene ciloleucel as second-line therapy for Large b-cell lymphoma. New Engl J Med (2022) 386:640–54. doi: 10.1056/NEJMOA2116133/SUPPL_FILE/NEJMOA2116133_DATA-SHARING.PDF 34891224

[46] De Matteis S Dicataldo M Casadei B Storci G Laprovitera N Arpinati M. Peripheral blood cellular profile at pre-lymphodepletion is associated with CD19-targeted CAR-T cell-associated neurotoxicity. Front Immunol (2023) 13:1058126/BIBTEX. doi: 10.3389/FIMMU.2022.1058126/BIBTEX 36726971 PMC9886226

[47] Yi D Gergis M Elgohary G Hsu J Yang Y Bi X. Chimeric antigen receptor T-cell therapies in lymphoma patients with central nervous system involvement. Hematol Oncol Stem Cell Ther (2022) 15 (3):66–72. doi: 10.56875/2589-0646.1024 36537908

[48] Lamure S van Laethem F de Verbizier D Lozano C Gehlkopf E Tudesq JJ. Clinical and product features associated with outcome of dlbcl patients to cd19-targeted car t-cell therapy. Cancers (Basel) (2021) 13:4279. doi: 10.3390/CANCERS13174279/S1 34503088 PMC8428364

[49] Seo W Jerin C Nishikawa H. Transcriptional regulatory network for the establishment of CD8+ T cell exhaustion. Exp Mol Med (2021) 53:202–9. doi: 10.1038/S12276-021-00568-0 PMC8080584 33627794

[50] Wherry EJ. T Cell exhaustion. Nat Immunol (2011) 12:492–9. doi: 10.1038/ni.2035 21739672

[51] Liu C Qi T Milner JJ Lu Y Cao Y. Speed and location both matter: Antigen stimulus dynamics controls CAR-T cell response. Front Immunol (2021) 12:748768/BIBTEX. doi: 10.3389/FIMMU.2021.748768/BIBTEX 34691062 PMC8531752

[52] Eyquem J Mansilla-Soto J Giavridis T van der Stegen SJC Hamieh M Cunanan KM. Targeting a CAR to the TRAC locus with CRISPR/Cas9 enhances tumour rejection. Nat 2017 543:7643 (2017) 543:113–7. doi: 10.1038/nature21405 PMC5558614 28225754

[53] Beider K Itzhaki O Schachter J Grushchenko-Polaq AH Voevoda-Dimenshtein V Rosenberg E. Molecular and functional signatures associated with CAR T cell exhaustion and impaired clinical response in patients with b cell malignancies. Cells (2022) 11:1140. doi: 10.3390/CELLS11071140/S1 35406703 PMC8997745

[54] Eskelinen EL. Roles of LAMP-1 and LAMP-2 in lysosome biogenesis and autophagy. Mol Aspects Med (2006) 27:495–502. doi: 10.1016/J.MAM.2006.08.005 16973206

[55] Aktas E Kucuksezer UC Bilgic S Erten G Deniz G. Relationship between CD107a expression and cytotoxic activity. Cell Immunol (2009) 254:149–54. doi: 10.1016/J.CELLIMM.2008.08.007 18835598

[56] Betts MR Brenchley JM Price DA de Rosa SC Douek DC Roederer M. Sensitive and viable identification of antigen-specific CD8+ T cells by a flow cytometric assay for degranulation. J Immunol Methods (2003) 281:65–78. doi: 10.1016/S0022-1759(03)00265-5 14580882

[57] Krzewski K Gil-Krzewska A Nguyen V Peruzzi G Coligan JE. LAMP1/CD107a is required for efficient perforin delivery to lytic granules and NK-cell cytotoxicity. Blood (2013) 121:4672–83. doi: 10.1182/BLOOD-2012-08-453738 PMC3674668 23632890

