# Supplementary material for: Monitoring of kinetics and exhaustion markers of circulating CAR-T cells as early predictive factors in patients with B-cell malignancies
Source: Front Immunol. 2023 Apr 14;14:1152498. doi: 10.3389/fimmu.2023.1152498 (PMC10140355; doi:10.3389/fimmu.2023.1152498)
Supplement: Supplementary file 1 [file DataSheet_1.docx]

Supplementary Material

Monitoring of kinetics and exhaustion markers of circulating CAR-T cells as early predictive factors in patients with B-cell malignancies

Clara Beatriz García-Calderón^1†^, Belén Sierro-Martínez^1†^, Estefanía García-Guerrero^1*^, Luzalba Sanoja-Flores^1^, Raquel Muñoz-García^2^, Victoria Ruiz-Maldonado^1^, María Reyes Jimenez-Leon^1^, Javier Delgado-Serrano^1^, Águeda Molinos-Quintana^1^, Beatriz Guijarro-Albaladejo^1^, Inmaculada Carrasco-Brocal^1^, José-Manuel Lucena^2^, José-Raúl García-Lozano^2^, Cristina Blázquez-Goñi^1^, Juan Luis Reguera-Ortega^1^, María-Francisca González-Escribano^2^, Marta Reinoso-Segura^1^, Javier Briones^3^, José Antonio Pérez-Simón^1^, and Teresa Caballero-Velázquez^1^.

*** Correspondence:** Dr. Estefanía García-Guerrero: egarcia-ibis@us.es

# Supplementary Figures and Tables

## Supplementary Figures

**Supplementary Figure 1. Evaluation of the specificity of the detection reagents for the identification of academic CAR-T cells by flow cytometry.** Untransduced (UTD) CD4+ **A)** and CD8+ **B)** T cells were stained at the same time as academic CAR-T cells and specificity of different reagents is shown. Depicted are median and individual values of four independent experiments. P-values between the indicated groups were calculated using unpaired Mann-Whitney U-t tests.

**Supplementary figure 2. Evaluation of the specificity of the detection reagents for the identification of commercial CAR-T cells by flow cytometry. A)** Peripheral mononuclear cells of healthy donors were stained with CD19 proteins from Acrobiosystems or Miltenyi and specificity of reagents was measured. Depicted are median and individual values of four independent experiments. P-values between the indicated groups were calculated using unpaired Mann-Whitney U-t tests.

**Supplementary Figure 3. Commercial CD19 CAR-T cell expansion in the blood of patients with lymphoma. A)** Absolute count of CAR-T cells/uL by flow cytometry in the blood of patients with lymphoma excluding patients with B-ALL. **B)** CAR copies/cell in the blood of patients with lymphoma excluding patients with B-ALL. **C)** Percentage of CAR+ cells from CD3 population in the blood of patients post-infusion.

**Supplementary Figure 4. Comparison of the expansion dynamics of different commercial CD19 CAR-T cell products.** Absolute count of CAR-T cells by flow cytometry in patients infused with Tisa-cel **A)** or Axi-cel **B)**. CAR copies/cell of patients infused with Tisa-cel **C)** or Axi-cel **D). E)** Median peak CAR-T cells absolute count *(left)* and day of peak expansion *(right)* by flow cytometry was analyzed between Tisa-cel and Axi-cel. **F)** Median day of peak CAR copies/cell *(left)* and median day of peak expansion *(right)* by dPCR was analyzed comparing patients infused with Tisa-cel and Axi-cel. Depicted are median and individual values of forty-eight samples. P-values between the indicated groups were calculated using unpaired Mann-Whitney U-t tests. ***p<0.001

**Supplementary Figure 5. Immunophenotype characterization of non-modified T cells and CAR-T cells at the time of peak expansion in blood of patients with lymphoma. A)** T cell subsets were analyzed at the time of peak expansion in patients with lymphoma excluding ALL patients. **B)** Immunophenotypic characterization of non-transduced T cells and CAR-T cells at the time of peak expansion were analyzed in patients with lymphoma excluding ALL patients. Depicted are median and individual values of thirteen samples. P-values between the indicated groups were calculated using unpaired Mann-Whitney U-t tests. *p<0.05, **p<0.01, ***p<0.001

**Supplementary Figure 6. Immunophenotype characterization of non-modified T cells and CAR-T cells at the time of peak expansion in blood of patients infused with Tisa-cel vs Axi-cel. A)** CD4/CD8 ratio of non-modified T cells and CAR-T cells of patients infused with Tisa-cel or Axi-cel. **B)** T cell subsets were analyzed at the time of peak expansion in patients infused with either Tisa-cel or Axi-cel **C)** Immunophenotypic characterization of non-transduced T cells and CAR-T cells at the time of peak expansion were analyzed in patients infused with Tisa-cel or Axi-cel. Depicted are median and individual values of nine-teen samples. P-values between the indicated groups were calculated using unpaired Mann-Whitney U-t tests. *p<0.05, **p<0.01, ***p<0.001

**Supplementary Figure 7. Comparison of T cell subsets between leukapheresis and non-modified T and CAR-T cells at the time of peak expansion. A)** CD4/CD8 ratio at the moment of leukapheresis and at peak expansion. **B)** T cell subsets in CD4 and CD8 compartments at the leukapheresis and in non-modified T and CAR-T cells. Depicted are median and individual values of nineteen samples. P-values between the indicated groups were calculated using unpaired Mann-Whitney U-t tests. *p<0.05, **p<0.01, ***p<0.001

**Supplementary Figure 8. Quantitative determination of the TCR Vβ repertoire of human T lymphocytes by flow cytometry.** T cell repertoire was analyzed at the time of peak expansion in 10 patients in both **A)** T cells and **B)** CAR-T cells.

**Supplementary Figure 9. Correlation between CAR-T cell expansion in blood of patients with lymphoma and toxicity or response. A)** Correlation between area under the curve (AUC) by flow cytometry (*left)* or dPCR *(right)* and severity of CRS from patients with B-ALL and lymphoma. **B)** Correlation between AUC by flow cytometry *(left)* or dPCR *(right)* and incidence of ICANs from patients with B-ALL and lymphoma. **C)** Correlation between AUC by flow cytometry (*left)* or dPCR *(right)* and severity of CRS from patients with lymphoma excluding patients with B-ALL. **D)** Correlation between AUC by flow cytometry *(left)* or dPCR *(right)* and incidence of ICANs from patients and lymphoma excluding patients with B-ALL. Correlation between AUC by flow cytometry ***E)*** or CAR copies/cell **F)** and event free survival of all patients or excluding patients with B-ALL **G)** and **H).** Depicted are median and individual values of the values of forty-eight samples. P-values between the indicated groups were calculated using unpaired Mann-Whitney U-t tests. *p<0.05. Correlation studies were performed using Kaplan-Meier survival analysis.

**Supplementary Figure 10. Comparison of the toxicity and efficacy of Tisa-cel and Axi-cel products. A)** Incidence of severe CRS (grades 2-3) in patients infused with Tisa-cel or Axi-cel products. **B)** Incidence of the appearance of ICANs in patients infused with Tisa-cel or Axi-cel products. **C)** Event-free survival of patients infused with Tisa-cel or Axi-cel products. P-values between the indicated groups were calculated using Fisher exact test. **p<0.01

**Supplementary Table 1**

| **Antibody** | **Fluorochrome** | **Company** | **Reference** |
| --- | --- | --- | --- |
| EGFR | APC | Biolegend | 352906 |
| Protein L | FITC | Acrobiosystems | RPL-PF141 |
| CAR Detection Reagent | Biotin | Miltenyi | 130-129-550 |
| Streptavidin | PE | BD | 405203 |
| Human CD19 Protein | FITC | Acrobiosystems | CD9HF251 |
| CD3 | V500 | BD | 560770 |
| CD4 | Pacific Blue | Biolegend | 317429 |
| CD8 | APC-H7 | BD | 561423 |
| 7AAD | PercP | BD | 559925 |
| CD45 | V500 | BD | 653806 |
| CD3 | APC | BD | 345767 |
| CD62L | FITC | BD | 347443 |
| CD95 | PE | BD | 555674 |
| CD3 | PercPCy5.5 | BD | 332771 |
| CD45RO | PeCy7 | BD | 337168 |
| CD4 | APC | BD | 345771 |
| CD8 | APCH-7 | BD | 641400 |
| CD45RA | Red178 | BD | 752257 |
| CD27 | V510 | Biolegend | 302836 |
| CD45 | BV650 | BD | 563717 |
| CD8 | FITC | Cytognos | CYT-8F8 |
| CD56 | PE | BD | 345810 |
| CD3 | APC-H7 | BD | 641415 |
| CD16 | PeCy7 | BD | 557744 |
| CD123 | APC | Miltenyi | 130-113-322 |
| CD14 | APC-H7 | BD | 641394 |
| CD11c | Red718 | BD | 566933 |
| CD4 | V450 | BD | 560345 |
| CD45 | OC-515 | Cytognos | CYT-45OC |
| HLADR | BV711 | BD | 563696 |
| TIM3 | PE | RyD System | FAB2365P |
| Lag3 | PeCy7 | Biolegend | 369211 |
| PD1 | PercPCy5.5 | BD | 561273 |
| FasL | APC | Biolegend | 106609 |
| CD8 | APC-H7 | BD | 641400 |
| CD3 | Red718 | BD | 566955 |
| CD4 | BV605 | BD | 562658 |
| Ki67 | PE | BD | 556027 |
| CD25 | FITC | BD | 345796 |
| CD69 | BB700 | BD | 747520 |
| CD107a | PeCy7 | BD | 328617 |
| CD45 | BV605 | BD | 564047 |
| Beta Mark TCR Vb Repertoire Kit | -- | Beckman Coulter | IM3497 |

**Supplementary Table 1. Flow cytometry reagents for the validation of the detection and immune-phenotype characterization of academic and commercial CD19 CAR-T cells**
